# Sunlight-Driven Photocatalysis in Hydrothermally Coupled ZnO/Fe_3_O_4_ Heterostructures from Bioengineered Nanoparticles

**DOI:** 10.3390/nano15241864

**Published:** 2025-12-11

**Authors:** Nayane O. Chaves, Michael D. S. Monteiro, Thayna M. Lira, Daniela B. Santos, Victor M. Del Aguila, Ștefan Țălu, Nilson S. Ferreira, Henrique Duarte da Fonseca Filho, Eliana M. Sussuchi, Rosane M. P. B. Oliveira, Robert S. Matos

**Affiliations:** 1 Postgraduate Program in Materials Science and Engineering (P^2^CEM), Federal University of Sergipe, São Cristovão 49100-000, SE, Brazil; nayaneochaves@academico.ufs.br (N.O.C.); thaynamlira@academico.ufs.br (T.M.L.); 2 Laboratory of Corrosion and Nanotechnology (LCNT), Federal University of Sergipe, São Cristovão 49100-000, SE, Brazil; michaelquimica96@gmail.com (M.D.S.M.); midori@academico.ufs.br (E.M.S.); 3 Amazonian Materials Group, Department of Physics, Federal University of Amapá, Macapá 68903-419, AP, Brazil; daniellasanttos77@gmail.com (D.B.S.); vmagui@unifap.br (V.M.D.A.); 4 The Directorate of Research, Development and Innovation Management (DMCDI), The Technical University of Cluj-Napoca, Constantin Daicoviciu Street, No. 15, 400020 Cluj-Napoca, Romania; stefan.talu@auto.utcluj.ro; 5 Department of Physics, Federal University of Sergipe, São Cristovão 49100-000, SE, Brazil; nilson@academico.ufs.br; 6 Laboratory for Development and Applications of Amazon Nanomaterials (LADENA), Department of Materials Physics, Federal University of Amazonas, Manaus 69067-005, AM, Brazil; hdffilho@ufam.edu.br

**Keywords:** green synthesis, ZnO/Fe_3_O_4_ heterostructure, sunlight-driven photocatalysis, defect-engineered interfaces, magnetic recyclability

## Abstract

We report a fully biogenic route to ZnO, Fe_3_O_4_, and their hydrothermally coupled ZnO/Fe_3_O_4_ heterostructure and establish a synthesis–structure–function link. Phase-pure, quasi-spherical wurtzite ZnO and finer inverse-spinel Fe_3_O_4_ nanoparticles assemble into a biphasic interface without forming a solid solution; optical analysis yields E_g_ = 2.36 eV (ZnO), 1.46 eV (Fe_3_O_4_), and 1.45 eV (ZnO/Fe_3_O_4_), while PL shows near-band-edge quenching and green–yellow defect reweighting at 490–560 nm, consistent with interfacial band bending. Magnetically, ZnO/Fe_3_O_4_ is soft-ferrimagnetic with M_S_/M_R_/H_C_ = 226 emu g^−1^/17 emu g^−1^/0.010 T (at 300 K), enabling rapid magnetic recovery. Under natural sunlight (572.6 ± 32 W m^−2^), adsorption-corrected methylene blue removal (10 mg L^−1^; 10 mg in 50 mL) gives real degradation rates RDR = 90% (ZnO), 65% (ZnO/Fe_3_O_4_), and 30% (Fe_3_O_4_) at 180 min, with pseudo–first-order constants k = 1.9 × 10^−2^, 0.7 × 10^−2^, and 0.4 × 10^−2^ min^−1^, respectively; dark adsorption baselines are 10%, 14%, and 49%. Reusability over four cycles preserves pseudo-first-order kinetics (ZnO/Fe_3_O_4_: 65% → 50%). Scavenger tests implicate OH• as the dominant oxidant in ZnO and ZnO/Fe_3_O_4_, and O2−• in Fe_3_O_4_. Taken together, the band alignment, photoluminescence quenching, radical-scavenger profiles, and kinetic synergy are consistent with a defect-rich S/Z-scheme-like ZnO/Fe_3_O_4_ heterojunction, delivering a green, sunlight-operable, and recyclable platform for affordable wastewater remediation.

## 1. Introduction

Sunlight-driven heterogeneous photocatalysis is a practical branch of advanced oxidation processes (AOPs) for degrading recalcitrant water pollutants because it converts photon energy directly into reactive oxygen species (ROS) capable of mineralizing organics to benign end products. In parallel, green synthesis has matured into a credible manufacturing route for metal-oxide nanomaterials, replacing harsh reductants and surfactants with plant-derived phytochemicals (polyphenols, flavonoids, terpenoids, sugars) that chelate metal ions, reduce precursors, and cap nascent crystallites, lowering chemical/energy footprints while encoding hydrophilicity and defect-rich surfaces beneficial to catalysis [1,2]. Contemporary reviews emphasize rigorous protocols, dark adsorption controls, validated kinetic windows, and realistic irradiance reporting, to avoid overstated performance and enable cross-study comparison under real or simulated sunlight [3,4]. Together, these trends converge on biogenic oxide photocatalysts that operate under genuine solar flux, report adsorption-corrected rates, and demonstrate multi-cycle reusability [5,6,7].

A recurring design principle is to pair a wide-gap photon harvester with a magnetic ferrite to obtain catalysts that can be separated by a hand magnet and reused without pressure filtration, an operational advantage that limits secondary nanoparticle pollution [8,9]. In such hybrids, Fe_3_O_4_ (inverse spinel, mixed Fe^2+^/Fe^3+^) supplies soft-magnetic response and redox plasticity, while a photoactive partner (e.g., ZnO) harvests UV/near-UV photons and initiates ROS; the heterointerface, its band offsets, internal fields, structural coherence, and defect landscape, governs charge routing and recombination suppression. Recent sunlight/visible-light studies showcase this strategy across architectures such as Fe_3_O_4_@ZnO core–shells [10,11], ZIF-derived Fe_3_O_4_/ZnO [12,13], and ternaries like Fe_3_O_4_@ZnO@Bi_2_O_2.7_ [14], consistently highlighting recyclability with sustained activity over multiple cycles. Biogenic/sol–gel routes are particularly attractive for such hybrids because residual organic caps can improve dispersion and seed vacancy-rich skins that favor interfacial H_2_O/O_2_ activation, while the magnetic ferrite ensures easy post-reaction recovery [14,15,16].

Among earth-abundant oxides, ZnO remains a benchmark photocatalyst for methylene blue (MB) abatement thanks to its low toxicity, scalable synthesis, strong band-edge absorption, and versatile defect chemistry [17,18,19]. Its optical and defect landscape can be rationally tuned by morphology control, dopant incorporation, and surface functionalization, enabling efficient harvesting of near-UV photons and activation of OH^−^/H_2_O into highly oxidizing ROS. Across morphologies and dopant sets, ZnO frequently displays pseudo-first-order kinetics under sunlight once dark adsorption is corrected, making it particularly amenable to kinetic benchmarking and cross-study comparison [20,21]. Several recent studies report rapid, reproducible MB degradation with ZnO or ZnO-based composites at realistic loadings, near-neutral pH, and modest light intensities, reinforcing its role as a standard against which more complex hybrids are measured [22,23,24]. Further, recent eco-sustainable routes have also shown that defect-rich ZnO nanoparticles obtained with natural extracts can simultaneously exhibit strong UV–vis absorption, intense defect-related PL and high photocatalytic and antimicrobial activity, particularly for methylene blue and other organic pollutants [25]. Conversely, Fe_3_O_4_ alone is generally adsorption-biased and photochemically weak for MB under sunlight; intervalence charge-transfer transitions and fast non-radiative relaxation depress ROS yields unless H_2_O_2_ is added (photo-Fenton regime) or the ferrite is hybridized with a more photoactive phase [25,26,27]. Hence, ZnO/Fe_3_O_4_ heterostructures often exhibit intermediate or ZnO-like kinetics but markedly superior operability due to magnetic retrieval, with absolute rates governed by interface quality, defect alignment, and optical cross-section dilution across the two phases [26,27,28,29].

Across oxide photocatalysts, oxygen vacancies and related defect ladders regulate absorption tails (Urbach energy), charge separation, and interfacial redox. For ZnO, vacancy-engineered surfaces accelerate hole trapping at –OH/H_2_O, tune band-edge positions relative to ROS potentials, and extend carrier lifetimes, diverting near-band-edge radiative channels into productive chemistry [30,31,32]. Correlative spectroscopy frequently links near-band-edge (NBE) quenching and green–yellow PL reweighting to oxygen vacancy states, which track enhanced ROS budgets and faster MB decay under irradiation [20,33,34]. Meanwhile, S-/Z-scheme junctions that exploit asymmetric defect landscapes and built-in fields can preserve high-energy carriers (holes on ZnO to generate OH•; electrons on Fe_3_O_4_ to produce O2−•) while annihilating low-energy ones, suppressing back-recombination and sustaining pseudo-first-order kinetics. These mechanistic pictures are reinforced by recent reviews and case studies highlighting defect-tailored ZnO and S-scheme heterostructures as robust platforms for dye photodegradation [35,36,37]. However, most reported systems still rely on energy-intensive or multistep syntheses, costly dopants, and limited recyclability, and only rarely integrate defect engineering, magnetic recovery, and genuinely low-cost green chemistry into a single design. Typical ZnO-based photocatalysts are obtained via hydrothermal, solvothermal, or calcination routes that demand high temperatures, long dwell times, or organic surfactants, undermining claims of sustainability [38,39,40]. Green routes based on plant extracts are emerging, but they often stop at demonstrating “eco-friendly” synthesis without quantitatively linking phytochemical composition to defect populations, band-structure tuning, or ROS budgets. In parallel, many Fe_3_O_4_/ZnO heterostructures exploit magnetic separability, yet their synthesis depends on separate, non-biogenic steps and rarely targets vacancy engineering as an explicit design variable [9,41]. Recyclability is commonly assessed over only a few cycles, with weak control of pH, catalyst loading, and solar flux, making cross-study benchmarking difficult. Thus, there is still a lack of integrated platforms where bio-directed defect control, magnetic retrieval, and sunlight-operable performance are co-optimized and mechanistically correlated.

Here, we elucidate how oxygen-vacancy landscapes govern photocatalytic response by exploiting *Maytenus rigida* Mart., a tannin- and triterpene-rich tropical plant [42,43], as a multifunctional phytochemical reservoir for the low-temperature, low-cost synthesis of defect-rich ZnO and Fe_3_O_4_ nanoparticles. These bio-derived building blocks are then integrated into a magnetically retrievable ZnO/Fe_3_O_4_ nanocomposite that couples vacancy-mediated band-structure tuning with S/Z-scheme-like charge flow, yielding high photocatalytic activity under sunlight together with effortless magnetic recovery and reuse. In recent years, S-scheme and direct Z-scheme heterojunctions have emerged as powerful architectures to reconcile efficient charge separation with strong redox ability, particularly in ZnO-based systems for pollutant degradation; in such junctions, interfacial band bending and internal electric fields drive recombination of low-energy carriers while preserving the most reducing electrons and most oxidizing holes in the extreme bands, thereby enhancing O2−•/OH• generation compared with conventional type-II layouts [35,44,45]. Motivated by this framework, we investigate whether our green-synthesized ZnO/Fe_3_O_4_ can be described as an S/Z-scheme-like heterojunction, using band-gap/edge analysis, photoluminescence and radical-scavenger experiments to build a mechanistic picture in analogy with reported ZnO-based S-scheme and Z-scheme photocatalysts [9,46,47,48]. By quantitatively correlating structural, optical and spectroscopic signatures of defects with ROS generation and MB degradation kinetics, our integrated strategy directly links nanomaterial defect landscapes to scalable, sustainable fabrication, establishing a practical blueprint for earth-abundant, green-engineered photocatalysts that simultaneously address performance, recyclability and synthetic simplicity.

## 2. Materials and Methods

### 2.1. Materials and Chemicals

A hydroethanolic extract of *M. rigida* was prepared following a previously established protocol [43] and served as the reducing and stabilizing agent for the synthesis of zinc oxide and magnetite nanoparticles. Hexamethyldisiloxane (HDMSO, ≥99%, Sigma-Aldrich, St. Louis, MO, USA) was used to dissolve the extract, and sodium hydroxide (NaOH, ≥98%, Sigma-Aldrich, St. Louis, MO, USA) was employed to adjust the solution pH. Zinc nitrate hexahydrate (Zn(NO_3_)_2_·6H_2_O, ≥99%, Sigma-Aldrich, St. Louis, MO, USA) was the ZnO precursor, while iron(II) sulfate heptahydrate (FeSO_4_·7H_2_O, ≥99%, Sigma-Aldrich, St. Louis, MO, USA) and iron(III) chloride hexahydrate (FeCl_3_·6H_2_O, ≥99%, Sigma-Aldrich, St. Louis, MO, USA) were used to generate Fe_3_O_4_. Photocatalytic tests used methylene blue (MB, PA, ≥99%, Neon Comercial, São Paulo, Brazil). Reactive-species scavenging assays employed isopropanol (IPA, ≥99.5%, Sigma-Aldrich, St. Louis, MO, USA), p-benzoquinone (BQ, ≥98%, Sigma-Aldrich, St. Louis, MO, USA), and disodium ethylenediaminetetraacetate dihydrate (EDTA, ≥99%, Sigma-Aldrich, St. Louis, MO, USA).

### 2.2. Synthesis of ZnO Nanoparticles

In a simple protocol, 0.1 g of *M. rigida* powder extract was dissolved in 10 mL of 1% HDMSO solution under continuous stirring until fully homogenized. Next, 1.0 g of Zn(NO_3_)_2_·6H_2_O was added and the mixture was stirred for 30 min. The resulting material was dried in an oven at 100 °C for 24 h and subsequently calcined in a muffle furnace at 400 °C for 4 h, yielding highly crystalline ZnO nanoparticles.

### 2.3. Synthesis of Fe_3_O_4_ Nanoparticles

In a typical procedure, 1.0 g of FeSO_4_·7H_2_O and 2.0 g of FeCl_3_·6H_2_O were dissolved in distilled water (50 mL) and heated to 80 °C. Then, 10 mL of the *M. rigida* solution was added and the mixture was stirred until homogeneous. The pH was adjusted to 11 with 1 M NaOH and stirring continued for 30 min. The suspension was allowed to cool to room temperature, and the Fe_3_O_4_ nanoparticles were magnetically separated, washed three times with distilled water, and dried at 80 °C for 12 h.

### 2.4. Synthesis of ZnO/Fe_3_O_4_ Nanocomposite

The ZnO/Fe_3_O_4_ nanocomposite (1:1 *w*/*w*) was prepared by a hydrothermal method. This equimass proportion was chosen *a priori* as a pragmatic compromise between three requirements: (i) preserving a sufficiently high ZnO fraction to sustain strong light harvesting and defect-mediated photocatalysis under sunlight, (ii) incorporating enough Fe_3_O_4_ to ensure efficient magnetic separation at low overall catalyst loading, and (iii) keeping the structural and mechanistic analysis focused on a single, well-defined composition. Similar Fe_3_O_4_–ZnO systems reported in the literature, e.g., [48,49,50] show that increasing the magnetic fraction enhances recoverability but progressively dilutes the photoactive ZnO phase. Initially, the ZnO nanoparticles (150 mg) were dispersed in distilled water (30 mL) using an ultrasonic bath for 10 min. The Fe_3_O_4_ nanoparticles (150 mg) were then added, and sonication was continued for an additional 10 min. The suspension was transferred to a Teflon-lined stainless-steel autoclave and heated at 180 °C for 18 h. The resulting precipitate was collected by centrifugation (4000 rpm, 10 min), washed three times with distilled water and ethanol, and dried at 80 °C for 12 h to obtain the final powder.

### 2.5. Characterization

Thermogravimetric analyses (TGA/DTG) were carried out on a TA Instruments Discovery TGA 55. Samples were heated from 25 to 1000 °C at 10 °C min^−1^ under N_2_ flow (60 mL min^−1^). Fourier-transform infrared (FTIR) spectra were acquired on a PerkinElmer Spectrum Two in the 4000–400 cm^−1^ range (2 cm^−1^ resolution, 20 scans) using KBr pellets. X-ray diffraction (XRD) patterns were collected on a Rigaku RINT PC D/MAX ULTIMA+ diffractometer with Cu Kα radiation (λ = 1.5406 Å), scanning 2θ = 10–80° (step size 0.02°, 0.4 s per step, 40 kV, 30 mA). Rietveld refinement was performed in FullProf using a modified pseudo-Voigt profile [51,52]; instrumental resolution was calibrated with LaB_6_, and peak profiles were inspected in WinPLOTR [53]. Line broadening followed the Caglioti formalism [54]; anisotropic size effects were modeled with real spherical harmonics (SPH) as a function of the diffraction vector [hkl] and Laue symmetry [51,55], while strain broadening was treated via quartic variance in reciprocal space with coefficients constrained by crystal symmetry. Morphology and selected-area electron diffraction (SAED) were examined by TEM (JEOL JEM-1400Plus, JEOL Ltd., Tokyo, Japan, operated at 120 kV). Photoluminescence (PL) spectra were recorded on a FP-8600 spectrometer (JASCO Corp., Tokyo, Japan), operating at 200–800 nm range. Magnetic properties were measured using a PPMS Dynacool (Quantum Design Inc., San Diego, CA, USA) with vibrating-sample magnetometry, acquiring M(H) at 50 and 300 K from −7 T to +7 T. The electrophoretic mobility and zeta potential of the samples were determined using a Zetasizer Advance ULTRA (Malvern Panalytical Ltd., Malvern, UK). The nanoparticles were dispersed in Milli-Q water containing 1 mM KCl, sonicated to ensure adequate homogenization, and the pH was adjusted to ~6.5–7.0 prior to analysis. Measurements were conducted at room temperature using disposable DTS1070 folded capillary cells. Electrophoretic mobility values were automatically converted to zeta potential by the ZS Xplorer 4.0.0 software using the Smoluchowski model.

### 2.6. Photocatalytic Evaluation

Photocatalytic activity of the nanoparticles and the nanocomposite was evaluated *via* MB degradation. In a typical assay, 10 mg of photocatalyst were dispersed in 50 mL of MB solution (10 mg L^−1^). The suspension was sonicated for 30 s to minimize agglomeration and then magnetically stirred for 5 min to homogenize. Dark controls were performed prior to irradiation to account for adsorption. For the irradiation experiment, the suspension was exposed to natural sunlight for 180 min (global irradiance 572.6 ± 32 W m^−2^, monitored with a digital lux meter, Metravi 1332; measurements between 11:00 and 14:00 during February–March in São Cristóvão, Sergipe, Brazil). At 20 min intervals, 3.5 mL aliquots were withdrawn and analyzed on a UV–Vis spectrophotometer (Varian Cary 100) at λ_max_ = 665 nm. The degradation rate (DR) was calculated according to Equation (1), where $A_{0}$ and A are the absorbances before and after sunlight irradiation, respectively, and $C_{0}$ and C are the corresponding dye concentrations. Kinetic analysis followed the Langmuir–Hinshelwood model, using $kt=-ln\left(\frac{C}{C_{0}}\right)$ to determine the apparent pseudo-first-order rate constant k, where t is the exposure time.

(1) $$DR\;(\%)=\left(\frac{C_{0}-C}{C_{0}}\right)\times 100\%=\left(\frac{A_{0}-A}{A_{0}}\right)\times 100\%$$

## 3. Results and Discussion

### 3.1. Chemical and Thermal Evaluation of the Products

**Figure 1:**
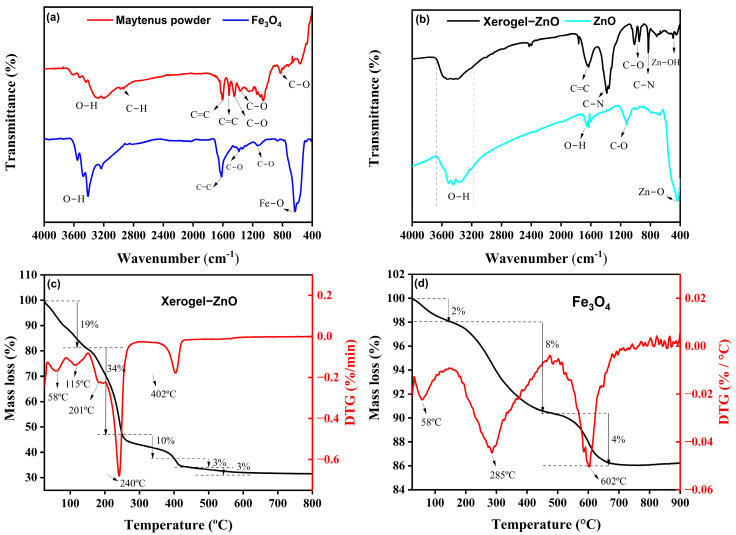
(**a**) FTIR spectra of *Maytenus rigida* powder and as-synthesized Fe_3_O_4_ nanoparticles. (**b**) FTIR spectra of xerogel precursor and ZnO nanoparticles, evidencing the disappearance of organic vibrations and emergence of the Zn–O stretching band upon crystallization. (**c**) TGA/DTG profiles of the xerogel precursor of ZnO nanoparticles. (**d**) TGA/DTG profiles of as-synthesized Fe_3_O_4_ nanoparticles.

The FTIR spectrum of the *Maytenus rigida* extract (Figure 1a) reveals a broad absorption band between 3500 and 3400 cm^−1^, attributed to the stretching vibrations of hydroxyl groups (–OH) in phenolic and alcoholic compounds [56]. The band near 2930 cm^−1^ corresponds to C–H stretching modes, typical of structures associated with proteins and carbohydrates [42]. Strong absorptions at 1604 and 1516 cm^−1^ indicate C=C stretching in aromatic rings, confirming the presence of tannins, flavonoids, and triterpenes [43]. Additional bands at 1366, 1060, and 825 cm^−1^ arise from C–O stretching vibrations related to functional groups such as ethers, alcohols, and carboxylic acids [42,57]. In contrast, the FTIR spectrum of the Fe_3_O_4_ nanoparticles (Figure 1a) shows substantial modification of these organic signatures, reflecting the chemical transformation mediated by phytochemical species during the reduction process. The broad bands at 3550, 3475, 3410, and 3234 cm^−1^ correspond to O–H stretching vibrations from residual surface hydroxyls [58]. The aromatic C=C stretching at 1618 cm^−1^ [59] and C–O vibrations at 1385 and 1136 cm^−1^ [60,61,62] indicate partial retention of organic residues from the extract acting as capping agents. Most notably, the emergence of a distinct absorption band at ~630 cm^−1^, assigned to Fe–O stretching [63], provides direct evidence for the formation of the spinel Fe_3_O_4_ crystalline phase. The spectral evolution from the complex organic fingerprint of *M. rigida* to the simplified metal–oxygen vibration of magnetite highlights the coordination of phytochemicals with Fe ions, promoting nucleation and stabilization of Fe_3_O_4_ nanoparticles under mild conditions. The FTIR spectra of the xerogel precursor and ZnO nanoparticles are presented in Figure 1b. The broad bands between 3500 and 3400 cm^−1^ correspond to O–H stretching, evidencing hydroxyl groups inherited from the plant extract [64]. In the xerogel, the low-frequency band at 689 cm^−1^ is assigned to Zn–OH bending vibrations [25], whereas the band at 510 cm^−1^ is characteristic of hydrozincite, Zn_5_(CO_3_)_2_(OH)_6_ [65,66], indicating the transient formation of basic zinc carbonate prior to oxide crystallization. Both spectra exhibit an absorption band near ~1630 cm^−1^, which can be attributed to C=C stretching vibrations in the xerogel [67] and to O–H bending (angular deformation) modes [65], while the bands at 1124 and 1015 cm^−1^ correspond to C–O stretching vibrations characteristic of alcohol groups [68]. Additional features at 1388 and 830 cm^−1^ correspond to C–N stretching vibrations [69]. After calcination at 400 °C, the appearance of a sharp band at 438 cm^−1^ in the ZnO spectrum confirms the establishment of Zn–O stretching modes [70], consistent with the wurtzite ZnO lattice.

The thermal stability and decomposition behavior of the xerogel precursor of ZnO nanoparticles, as well as the Fe_3_O_4_ nanoparticles were examined by TGA/DTG analyses in the temperature range of 25–1000 °C. The TGA/DTG curve of the xerogel precursor of ZnO nanoparticles (Figure 1c) exhibits three main stages of mass loss, revealing the sequential elimination of physically adsorbed and chemically bound species. The initial weight reduction of approximately 19% between 25 °C and 150 °C, with distinct DTG peaks at 58 °C and 115 °C, is attributed to the evaporation of physisorbed and weakly bound water molecules on the surface of Zn(OH)_2_ clusters and residual plant-derived organics [71]. In the subsequent region, from 150 °C to 250 °C, a more pronounced mass loss of 34% occurs, accompanied by two DTG thermal events at 201 °C and 240 °C, corresponding to the decomposition of residual organic matter and the release of volatile compounds originating from the *M. rigida* extract [72]. This step marks the progressive breakdown of biomolecular ligands that acted as chelating and stabilizing agents during synthesis. A third weight loss of 16%, extending from 250 °C to 600 °C and peaking near 402 °C, is associated with the oxidative degradation of remaining carbonaceous residues and the transition of amorphous intermediates to crystalline ZnO [72,73]. Beyond 400 °C, the absence of significant mass variation indicates the complete removal of organic moieties and the attainment of thermal stability of the oxide lattice, justifying 400 °C as the optimal calcination temperature. This multistage pattern, water release, organic decomposition, and oxide crystallization, is characteristic of plant-mediated ZnO systems and is widely reported in TGA/DTG profiles of green-synthesized ZnO nanoparticles, providing an external benchmark for the transitions observed here [74]. In addition, the conversion behavior of basic zinc carbonate/hydrozincite precursors corroborates the ~350–450 °C crystallization window toward wurtzite ZnO [75]. For Fe_3_O_4_ (Figure 1d), the TGA/DTG curves reveal markedly higher overall stability: a minor ~2% loss below 150 °C with a DTG event near ~58 °C is attributed to moisture removal [76], followed by ~8% loss between 150 and 480 °C (DTG at ~285 °C) due to decomposition of residual phytochemicals/capping groups from the extract [77]. A final ~4% decrement from ~480 to 725 °C, peaking near ~602 °C, is consistent with the oxidative conversion of magnetite to hematite (α-Fe_2_O_3_), in line with established oxidation pathways and temperature ranges for Fe_3_O_4_ → α-Fe_2_O_3_ transformations [78]. The smaller cumulative mass loss of the Fe_3_O_4_ sample compared to ZnO indicates a lower organic load and the intrinsic robustness of the spinel framework, aligning with its higher temperature onset for structural change [79].

### 3.2. Characterization of the Nanomaterials

**Figure 2:**
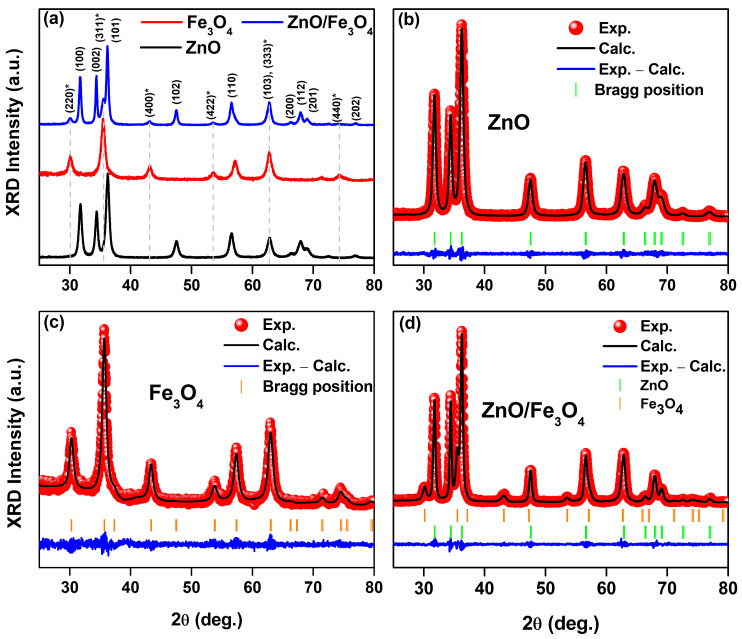
(**a**) Stacked powder XRD patterns of wurtzite ZnO (calcined at 400 °C), as-synthesized spinel Fe_3_O_4_, and the ZnO/Fe_3_O_4_ nanocomposite. (**b**–**d**) Rietveld refinements (red symbols, observed; black line, calculated; blue line, difference) with vertical ticks marking Bragg positions. The asterisk (*) indicates the reflection assigned to Fe_3_O_4_ spinel phase.

Figure 2a shows that the ZnO sample is phase-pure wurtzite (P6_3_mc), matching the reference pattern for hexagonal ZnO (ICSD card 76641). Further, the Fe_3_O_4_ pattern indexes to the inverse-spinel structure (Fd$\bar{3}$m; ICSD card 158745), with the characteristic (220), (311), (400), (422), (511), and (440) reflections. The nanocomposite shows both sets of Bragg reflections with no extra peaks within the detection limit, indicating a biphasic ZnO/Fe_3_O_4_ material without detectable spurious oxides. This assignment is coherent with the FTIR data: the composite retains the distinct lattice vibrations of each parent oxide (Zn–O and Fe–O bands) alongside the pronounced attenuation of organic bands after calcination, exactly as expected for a two-phase inorganic system stabilized by the bio-route. Rietveld refinements (Figure 2b,c) further confirm that the hydrothermal assembly (180 °C, 18 h) forms a biphasic ZnO/Fe_3_O_4_ heterostructure rather than a solid solution. The low residuals (Rwp = 8–11%) and χ^2^ = 1.1–1.24 indicate statistically robust models [80]. Crucially, the unit-cell metrics remain essentially invariant after composite formation, ZnO: a = b ~3.2538 → 3.2513 Å, c ~5.2130 → 5.2090 Å; Fe_3_O_4_: a ~8.3479 → 8.3840 Å, within typical refinement uncertainty for nanosized oxides [81]. The lack of systematic peak shifts in the composite fit rules out appreciable Zn–Fe interdiffusion at 180 °C and supports a clean heterointerface between wurtzite and spinel frameworks [82]. Line-profile parameters capture how the structure evolves at that interface. The apparent crystallite size of ZnO increases (D_XRD_ ~15.2 → 20.8 nm) while Fe_3_O_4_ remains ~8–9 nm; simultaneously, microstrain decreases in ZnO (ε: 41 → 24%) and increases in Fe_3_O_4_ (23 → 29%). A consistent picture is that Fe_3_O_4_ nanograins act as heterogeneous docking/anchoring sites, enabling partial coalescence or low-angle oriented attachment of adjacent ZnO domains [83,84], which relieves wurtzite lattice distortions. The converse, the modest ε increase in Fe_3_O_4_ is attributable to coherency/mismatch strain at the ZnO/Fe_3_O_4_ boundary where the oxygen sublattices meet with different stacking symmetries (hexagonal vs. cubic) [81,82]. This asymmetric size–strain partitioning aligns with reports for hydrothermal ZnO/Fe_3_O_4_ hybrids, where ZnO tends to coarsen slightly while spinel remains finely divided, yielding sharp two-phase refinements without peak drift [85,86]. Those studies likewise associate interface-mediated strain with enhanced charge separation and defect-state reconfiguration, features that later manifest in photocatalytic and (photo)magnetic responses of ZnO/Fe_3_O_4_ nanocomposites. The stable unit-cell volumes and near-reference densities in Table 1 further indicate that neither phase suffers oxygen non-stoichiometry beyond the norm for nanoscale wurtzite/spinel prepared under mild conditions [81], consistent with the phase-pure Bragg-mark patterns and smooth “Obs–Calc” residuals in Figure 2b–d. Thus, our refinements portray a mechanically and crystallographically intact heterostructure: ZnO undergoes modest coarsening with strain relief; Fe_3_O_4_ remains nanosized with slight strain buildup, an evolution fully consistent with low-temperature hydrothermal assembly of wurtzite–spinel couples.

**Figure 3:**
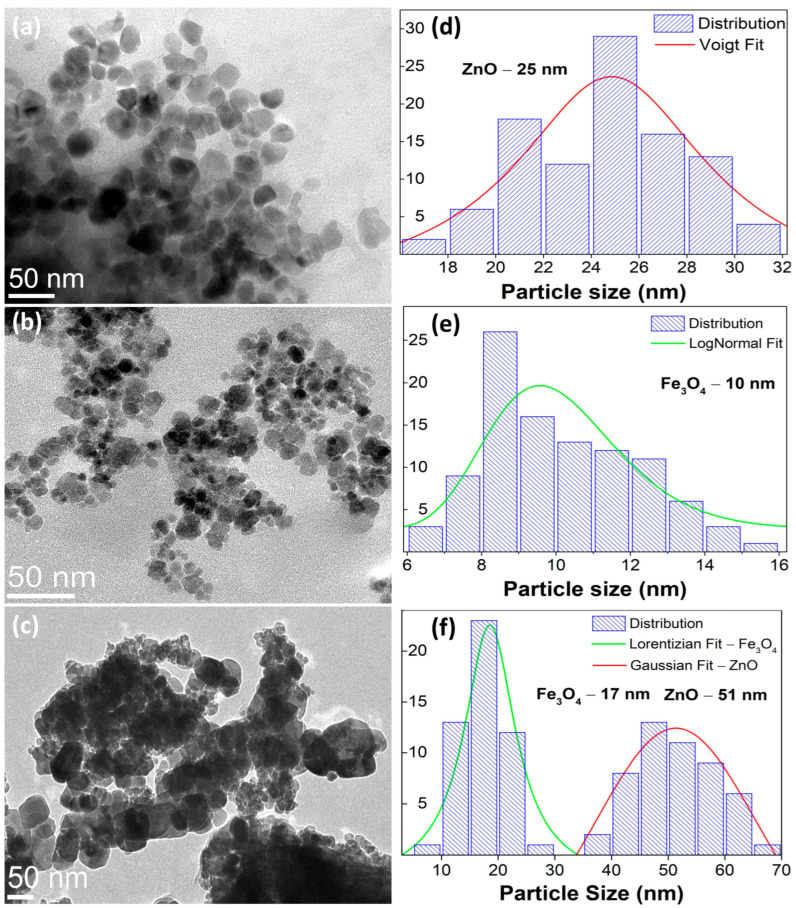
Bright-field TEM micrographs of wurtzite (**a**) ZnO, (**b**) inverse-spinel Fe_3_O_4_, and (**c**) the ZnO/Fe_3_O_4_ nanocomposite. (**d**–**f**) Particle-size distributions obtained from TEM (≥100 particles per sample) with model fits: ZnO (Voigt fit), Fe_3_O_4_ (log-normal fit), and the composite (Gaussian for ZnO and Lorentzian for Fe_3_O_4_).

**Figure 4:**
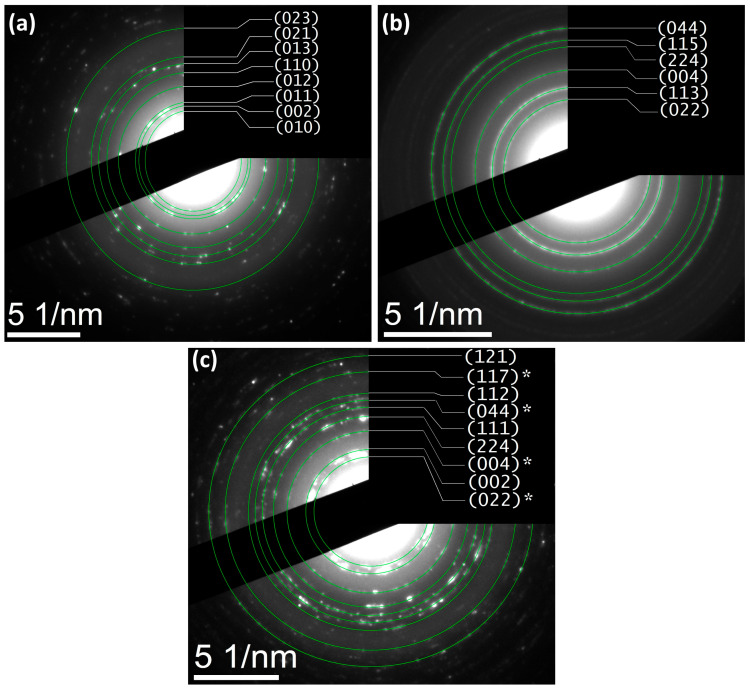
SAED patterns indexed to (**a**) ZnO (wurtzite), (**b**) Fe_3_O_4_ (inverse spinel), and (**c**) ZnO/Fe_3_O_4_ nanostructures, displaying concentric diffraction rings consistent with polycrystalline domains. Asterisks (*) mark reflections assigned to Fe_3_O_4_.

The morphological analysis (Figure 3a–c) reveals quasi-spherical ZnO domains (∼25 nm) and finer Fe_3_O_4_ nanoparticles (∼10 nm), while the hydrothermally coupled ZnO/Fe_3_O_4_ composite displays a heterogeneous assembly in which Fe_3_O_4_ nanograins decorate and bridge larger ZnO clusters. Particle-size statistics (≥100 particles, Figure 3d–f) show selective coarsening of ZnO (to ∼51 nm) and modest growth of Fe_3_O_4_ (to ∼17 nm). This asymmetric evolution is consistent with interfacial nucleation/coalescence of wurtzite domains on magnetite seeds under hydrothermal conditions and with the strain partitioning inferred from the XRD/Rietveld line-profile analysis (larger strain relaxation in ZnO, mild strain increase in Fe_3_O_4_). Comparable trends, ZnO growth with magnetite retained at smaller sizes and strong interfacial coupling, are frequently reported for Fe_3_O_4_/ZnO heterostructures synthesized at low temperature [9,49]. SAED patterns exhibit concentric rings that index to wurtzite-ZnO (Figure 4a) and inverse-spinel-Fe_3_O_4_ (Figure 4b), confirming polycrystalline character for both components and the biphasic nature of the composite (Figure 4c). The absence of extra rings or ring splitting is in line with the XRD two-phase refinement and the negligible lattice-parameter drift between the single-phase samples and the composite, i.e., no measurable solid-solution formation at 180 °C. Similar SAED signatures (distinct ZnO and Fe_3_O_4_ rings with clean superposition) are documented for Fe_3_O_4_/ZnO core–shell and coupled systems and used as a microstructural cross-check of phase purity [9,87]. These results provide a microstructural picture that dovetails with XRD/Rietveld: a crystallographically intact heterointerface between ZnO and Fe_3_O_4_, where ZnO coarsens slightly while Fe_3_O_4_ remains nanosized. This morphology is also compatible with reports that interfacial contact in Fe_3_O_4_/ZnO couples enhances carrier separation and limits uncontrolled agglomeration when processing windows are optimized [9].

**Figure 5:**
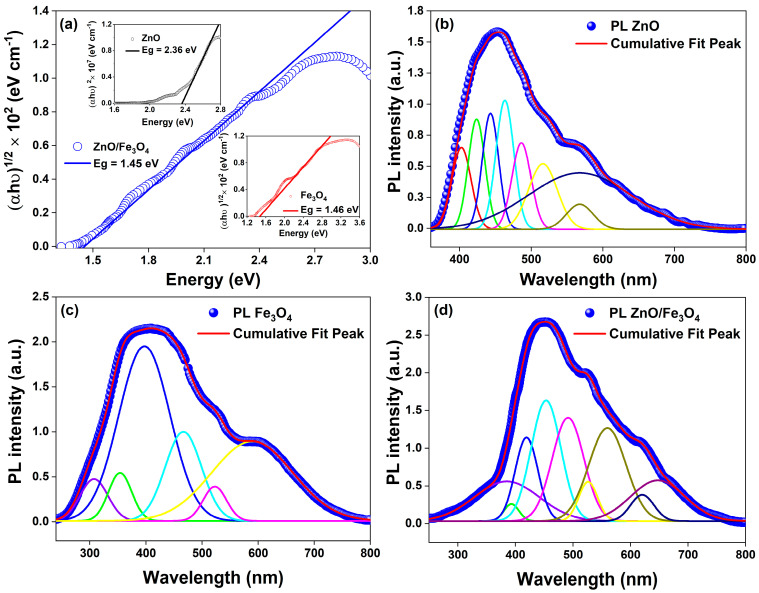
(**a**) Bandgap luminescence analysis for ZnO/Fe_3_O_4_ (E_g_ = 1.45 eV, main panel); insets: ZnO (E_g_ = 2.36 eV) and Fe_3_O_4_ (E_g_ = 1.46 eV). (**b**–**d**) Room-temperature deconvolved steady-state PL spectra for ZnO, Fe_3_O_4_, and ZnO/Fe_3_O_4_ nanomaterials.

Building on the structural and microstructural analysis, the optical response pivots accordingly: the interfacial microstrain and phase coupling evidenced for ZnO and Fe_3_O_4_ translate directly into band-edge reshaping and defect-state reweighting, as captured by the Tauc plots and room-temperature PL deconvolutions in Figure 5; specifically, we compute E_g_ = 2.36 eV for ZnO, 1.46 eV for Fe_3_O_4_, and 1.45 eV for the ZnO/Fe_3_O_4_ composite (Figure 4a), the latter indicating that the narrow-gap ferrite pins the low-energy absorption while ZnO supplies high-energy excitonic channels whose radiative weight is strongly modulated at the junction. For the green-synthesized ZnO, the Tauc analysis of the DRUV–vis data yields an apparent optical band gap of ~2.36 eV, which is lower than the canonical 3.2–3.3 eV reported for stoichiometric bulk ZnO. This is not inconsistent with the near-band-edge PL peaks at 399–423 nm (~3.1–3.2 eV), but rather reflects the strongly defected nature of our biogenic material. In heavily oxygen-vacancy–rich or plant-mediated ZnO nanostructures, several authors have reported similar band-gap narrowing and pronounced visible tails, with effective gaps in the 2.4–2.8 eV range while the excitonic PL remains close to the intrinsic band edge, attributing this *behaviour* to Urbach tails and dense sub-band-gap defect states associated with $V_{O}$, lattice strain and surface complexes [88,89,90]. In this framework, the lower E_g_ extracted from diffuse reflectance represents a defect-influenced optical threshold governed by band-to-tail and tail-to-band transitions into vacancy-derived states, whereas the NBE PL still probes recombination across a fundamental ZnO gap of ~3.2 eV. Consistent with this picture, our samples exhibit both a sharp UV emission and a broad green–yellow band (490–560 nm) assigned to $V_{O}$-related levels, together with a substantial sub-band-gap absorption tail seen in our bandgap luminescence analysis. Thus, the value of 2.36 eV should be understood as an apparent, defect-mediated optical gap of highly non-stoichiometric ZnO, rather than the intrinsic Γ–Γ band gap of ideal wurtzite ZnO, in line with the defect-engineering and green-synthesis literature cited above.

Our PL analysis shows that, in the single-phase ZnO, a weak NBE component at ~399 nm sits atop a multiband visible manifold extending from ~423 to ~567 nm (Table 2); consistent with recent defect spectroscopy in ZnO [91,92,93], we assign 423 nm to CB → $V_{Zn}$ and ${Zn}_{i}$ → VB transitions [94,95,96], 440–462 nm to donor–acceptor channels involving ${Zn}_{i}$/$O_{i}$/$V_{Zn}$ [97,98,99,100,101,102], and 487–567 nm to the oxygen-vacancy ladder ($V_{O}/V_{O}^{+}/V_{O}^{++}$) [97,100,103,104,105,106,107,108,109,110], including surface-ionized centers $V_{O}^{+/++}@S$ that dominate the green–yellow sector when the Fermi level is bent by surface or interfacial fields. These attributions align with current consensus that blue–green PL in ZnO is governed by intrinsic point defects whose charge state and energy are tuned by local strain and surface chemistry, trends consolidated in 2022–2024 studies that correlate the relative intensities of the ~440–520 nm bands with $V_{O}/{Zn}_{i}$ populations and Urbach energy [110]. In Fe_3_O_4_, the broad and weaker emission spanning ~307–585 nm arises from intervalence/defect-assisted pathways in the inverse-spinel [111,112]; we resolve a near-edge, hole-assisted feature near 398 nm, intermediate mid-gap bands at 354–465 nm [55,113], and deeper centers at 520–585 nm [42,114,115,116] attributable to $V_{O}$ and $V_{Fe}$ (with $V_{O}$@S contributions), in line with recent observations of defect-enabled photoluminescence from ferrite nanostructures and Fe_3_O_4_-containing hybrids [113,114,115,116]. In the heterostructure, the spectrum retains fingerprints of both lattices but with two decisive changes that track carrier dynamics at the interface: first, the ZnO NBE (~380–399 nm) is markedly quenched, and second, green–yellow bands (∼490–560 nm) gain relative weight while new/stabilized interfacial features appear at 394–525 nm. We ascribe 380 nm to ${NBE}_{ZnO}$ [91,92,93,97] and 394 nm to an ${NBE}_{{Fe}_{3}O_{4}}$-like edge ($h^{+}$ pathway) [55,117]; 418 and 452 nm map onto ${Zn}_{i}\to VB$ and ${Zn}_{i}\to V_{Zn}$ donor–acceptor pairs now perturbed by built-in fields [94,95,96,118,119]; 490 and 525 nm arise from ($V_{O}/V_{O}^{+}/V_{O}^{++}$ (including ${\;V}_{O}^{+/++}@S$) enriched or stabilized at the junction [97,120,121]; 560 nm reflects deeper $V_{O}^{+}/V_{O}^{++}$ ladders [116,122,123]; and the red-edge features at 620–650 nm are consistent with $O_{i}$/$V_{Fe}$-assisted recombination [124,125,126,127,128]; the global effect, suppression of UV/blue NBE with survival/intensification of green–yellow bands, is the canonical PL signature of interfacial charge extraction in ZnO-based heterostructures [129,130,131]. This optical redistribution dovetails with the structural motif commonly reported for Fe_3_O_4_–ZnO heterostructures (core–shell or coupled grains): coherent to semi-coherent *contacts* (*e.g.*, *ZnO* wurtzite facets against {111} Fe_3_O_4_) introduce misfit strain and oxygen nonstoichiometry, boosting deep-trap density and the Urbach tail while creating internal fields that separate excitons before fast NBE recombination, precisely the scenario that yields PL quenching in the UV/blue and persistent radiative channels from vacancy ladders [10,49]. Mechanistically, recent band-alignment analyses for Fe_3_O_4_/ZnO place the conduction-band edges such that photoelectrons generated in the ferrite can transfer to ZnO while holes are retained or scavenged, producing an interfacial field and S-/Z-scheme-like carrier flow that reconciles (i) decreased near-edge radiative recombination, (ii) enhanced mid-gap emission from stabilized $V_{O}$-rich surface states, and (iii) the composite’s apparent gap being pinned near the ferrite edge; our Tauc and PL data follow this template and match the charge-transfer picture resolved in contemporary Fe_3_O_4_/ZnO photocatalyst studies [12]. In other words, the optical modifications between single nanoparticles and the nanocomposite can thus be explained as follows: gap pinning and tailing toward Fe_3_O_4_ (Figure 4a) that increases visible-light absorptance; NBE quench in ZnO signaling efficient exciton dissociation across the junction; defect-state reweighting (490–560 nm) consistent with a higher density/accessibility of $V_{O}$-type centers at the interface; and emergent interfacial lines (394–525 nm) that track strain-induced band bending and hybridized traps, an optical “fingerprint” also reported for ZnO heterostructures where microstructural coherence and interfacial dipoles were confirmed by XRD/TEM and linked quantitatively to PL evolution [10,110]. This combination of strong NBE quenching and selective reshaping of the visible defect band in ZnO/Fe_3_O_4_ is characteristic of defect-rich oxide–Fe_3_O_4_ interfaces [132] where interfacial band bending *favours* nonradiative transfer and spatial separation of carriers, rather than a mere physical mixture of two radiatively independent phases. Similar PL signatures (suppressed NBE emission and reweighted deep-level bands) have been reported as key diagnostic features of S-scheme or direct Z-scheme charge transfer in ZnO/g-C_3_N_4_, ZnO/BiVO_4_ and related heterostructures [47,133,134,135]. Thus, coupling ZnO to Fe_3_O_4_ reshapes the band landscape and defect thermodynamics so that radiative recombination migrates from excitonic/near-edge channels to vacancy-mediated ones, while the absorption edge shifts/red-weights into the solar-visible window, an interplay well documented and directly advantageous for sunlight-driven catalysis and photo(electro)chemical function [12,129,130].

**Figure 6:**
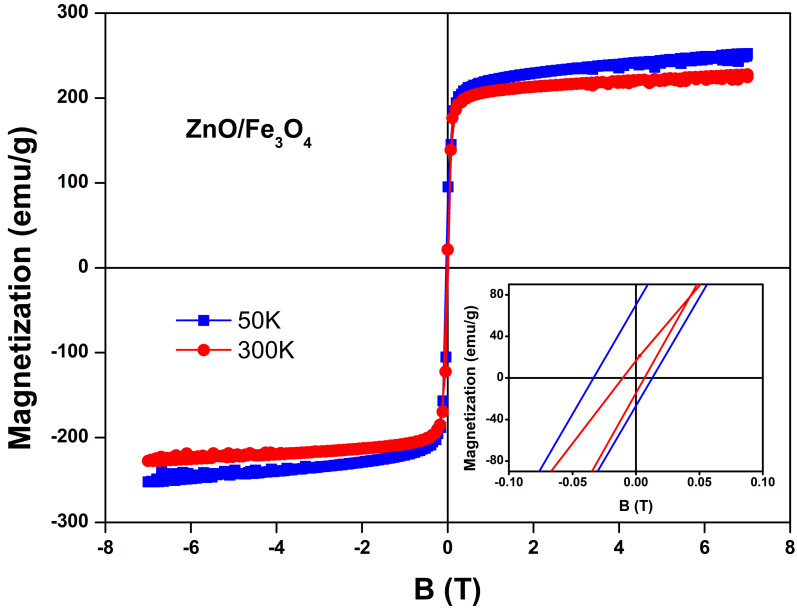
Field-dependent magnetization (M–B) curves of the ZnO/Fe_3_O_4_ nanocomposite measured at 50 K and 300 K. The inset highlights the enlarged low-field region, showing the coercive field and remanent magnetization differences between the two temperatures.

Consistent with the coherent ZnO–Fe_3_O_4_ coupling seen structurally, the M–B loops of the nanocomposite (Figure 6) display soft-ferrimagnetic hysteresis that tightens at low temperature: at 50 K we measure saturation magnetization M_S_ = 252 emu/g, remanent magnetization M_R_ = 70 emu/g and coercive field H_C_ = 0.034 T, while at 300 K the loop narrows to M_S_ = 226 emu/g, remanent magnetization M_R_ = 17 emu/g and coercive field H_C_ = 0.010 T. The large H_C_ and M_R_ at 50 K indicate blocked moments where the effective anisotropy barrier dominates the thermal energy; thermal agitation at 300 K averages surface/shape anisotropy and interparticle dipolar couplings, yielding the near-superparamagnetic response desirable for magnetic recovery, behavior widely reported for Fe_3_O_4_–oxide photocatalysts and Fe_3_O_4_ nanocomposites [9,14,136]. Interfacial oxygen-vacancy-rich regions and cation off-stoichiometry, from the optical section’s 490–560 nm PL (Table 2), promote surface-spin canting and interfacial anisotropy, thickening a magnetically disordered shell that raises H_C_ and M_R_ at low T; heating depins these canted spins, shrinking the loop, an effect resolved in recent studies on iron-oxide nanoparticles where surface spins dominate relaxation [137,138]. Coherent/semi-coherent ZnO/Fe_3_O_4_ contacts also inject microstrain and can alter Fe–O–Fe angles at antiphase boundaries, tuning superexchange and coercivity; controlling such defects is now recognized as a reliable handle on ferrite anisotropy [139,140]. The nanoparticle growth directly modulates this balance as follows: (i) as particles grow, the surface-to-volume ratio falls, the canted-spin shell thins relative to the ordered core, and M_S_ tends to increase while H_C_ can decrease due to reduced surface anisotropy; (ii) growth across the single-domain window can either raise H_C_ (approaching the single-domain size) or lower it once multi-domain reversal becomes favorable; (iii) improved crystallinity during growth reduces antiphase-boundary density and spin-disordered shells, further pushing M_S_ toward bulk-like values and softening room-temperature loops; (iv) larger volumes raises the blocking temperature (T_B_ ∝ K_eff_V), explaining the stronger remanence at 50 K for the larger, better-ordered crystallites. These size/defect trends are consistent with single-particle magnetometry on 20 nm Fe_3_O_4_, with spectroscopy that isolates surface vs. bulk spin dynamics, and with reviews linking surface canting to reduced M_S_ in ultrasmall grains [141,142,143]. Finally, growth also reshapes the interface with ZnO: coarser Fe_3_O_4_ grains reduce specific interfacial area and the density of strain-stabilized $V_{O}$ traps (those that intensified the 490–560 nm PL), weakening interfacial anisotropy; conversely, finer grains maximize interfacial defect density, reinforcing low-T H_C_/M_R_ and the PL-visible fingerprint. Recent Fe_3_O_4_–ZnO hybrids report the same co-variation between size-controlled interfaces, superparamagnetic-like room-T loops (for magnetic recovery), and retained photocatalytic activity, precisely the co-design targeted here [49].

### 3.3. Photocatalytic Analysis

**Figure 7:**
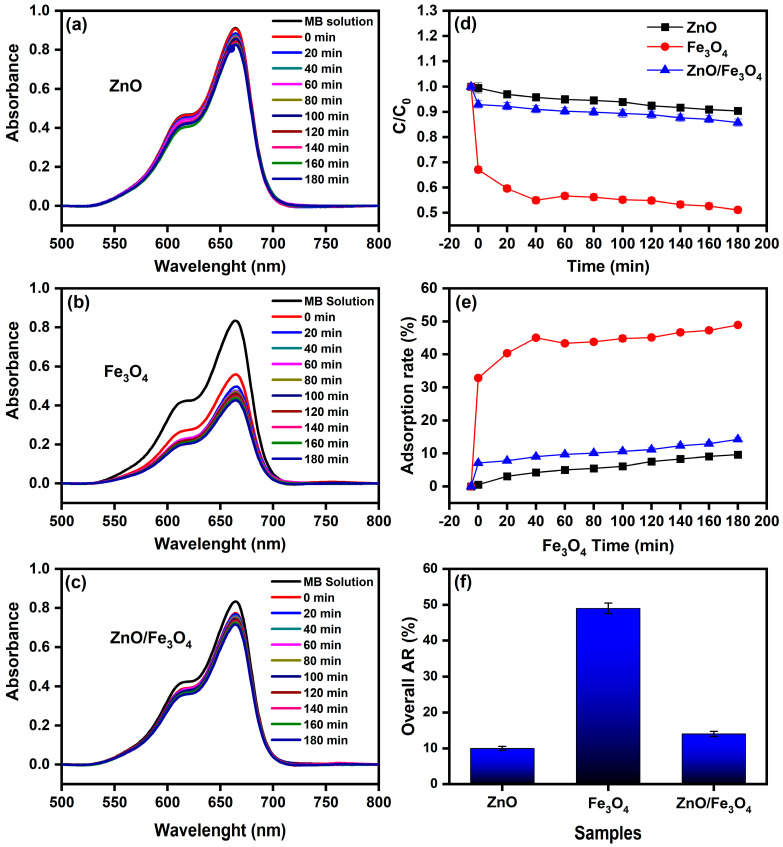
(**a**–**c**) UV–vis spectra of MB solution (10 mg L^−1^) recorded from 0 to 180 min in the absence of light using ZnO, Fe_3_O_4_, and ZnO/Fe_3_O_4_ nanomaterials; (**d**) Normalized concentration (C/C_0_) versus time; (**e**) Instantaneous adsorption rate; (**f**) Overall adsorption removal (AR) after 180 min.

**Figure 8:**
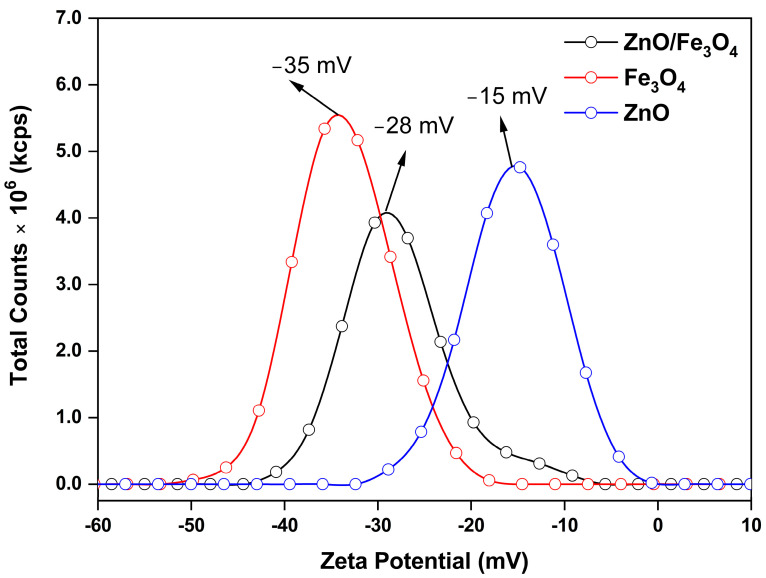
Zeta potential distributions of ZnO, Fe_3_O_4_, and ZnO/Fe_3_O_4_ nanomaterials measured in aqueous suspension.

Here, we assess the photocatalytic activity of the ZnO, Fe_3_O_4_, and ZnO/Fe_3_O_4_ powders toward MB using a protocol that cleanly separates dark adsorption from light-driven degradation. To avoid conflating sorption with catalysis, we first quantified the adsorption baseline in the absence of light (control experiment, CE; Figure 7a–f), extracting instantaneous rates and overall adsorption removal (AR). These CE values are then used to correct all sunlight measurements, so that we report the real degradation rate (RDR = DR − AD) and the corresponding pseudo-first-order constants. This two-step approach allows us to compare intrinsic reaction efficiencies across materials, identify the specific role of Fe_3_O_4_ in dark uptake, and determine how the heterointerface modifies the fate of MB under illumination. Under strictly dark conditions (Figure 7a–c), the concentration decay reflects adsorption rather than photochemistry. The trend Fe_3_O_4_ ≫ ZnO/Fe_3_O_4_ > ZnO in C/C_0_ (Figure 7d) and overall AR after 180 min (Figure 7f) is quantified by AD = 49% (Fe_3_O_4_), 14% (ZnO/Fe_3_O_4_) and 10% (ZnO). This hierarchy is consistent with the zeta-potential distributions in Figure 8, where Fe_3_O_4_, ZnO/Fe_3_O_4_ and ZnO exhibit ζ ≈ −35, −28 and −15 mV, respectively. Because MB is a cationic dye, more negative ζ values enhance electrostatic attraction between the chromophore and the particle surface, promoting higher surface coverage. Magnetite-rich surfaces thus provide both abundant –Fe–OH sites and the most favorable electrostatic environment, explaining their high dark adsorption and fast approach to equilibrium, in agreement with previous reports on Fe_3_O_4_-based sorbents [144,145]. In contrast, ZnO displays weak dark uptake, which is expected near neutral pH because its point of zero charge is relatively high; under these conditions the ZnO surface is partially protonated (${ZnOH}_{2}^{+}$) and tends to repel cationic MB, suppressing adsorption [5,26]. The heterostructure shows intermediate AR, attributable to (i) reduced exposed Fe_3_O_4_ area when coated or partially covered by ZnO and (ii) interfacial modification of Fe_3_O_4_ hydroxyl groups, which moderates the strong MB affinity of bare magnetite, a behavior also reported for Fe_3_O_4_–ZnO and related magnetic composites [146,147]. The instantaneous adsorption rates (Figure 7e) mirror these site-density/electrostatic arguments: Fe_3_O_4_ rapidly reaches a high-coverage plateau, whereas ZnO adsorbs slowly and sparsely, with the composite lying between these limiting cases.

**Figure 9:**
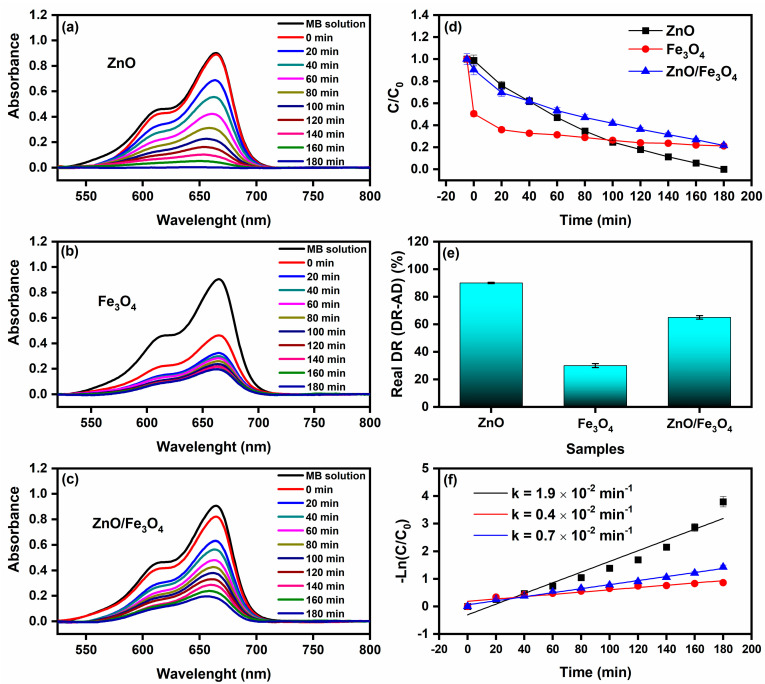
Sunlight-driven methylene blue (MB, 10 mg L^−1^) degradation using (**a**) ZnO, (**b**) Fe_3_O_4_, and (**c**) ZnO/Fe_3_O_4_ as photocatalysts. (**d**) Normalized concentration C/C_0_ versus time. (**e**) Real degradation rate (DR–AD), computed as degradation rate under sunlight (DR)-dark adsorption rate (AD). (**f**) Pseudo-first-order kinetic fit of the reaction as a function of irradiation time.

After subtracting dark uptake (CE), all trends in Figure 9 reflect true photo-removal of MB. The normalized profiles C/C_0_ (Figure 9d) show a rapid drop for ZnO, intermediate decay for ZnO/Fe_3_O_4_, and the slowest decline for Fe_3_O_4_, consistent with the RDR values in Table 3: ZnO = 90%, ZnO/Fe_3_O_4_ = 65%, Fe_3_O_4_ = 30% after 180 min. Kinetic fits to −ln(C/C_0_) = kt (Figure 9f) yield k_ZnO_ = 1.9 × 10^−2^ min^−1^, k_ZnO/Fe3O4_ = 0.7 × 10^−2^ min^−1^, and k_Fe3O4_ = 0.4 × 10^−2^ min^−1^. The ordering matches contemporary sunlight/visible-light MB studies, where ZnO typically follows pseudo–first-order kinetics and outperforms bare iron oxides under comparable doses, while magnetic hybrids sit between the two depending on interface quality and optical cross-section [27,148]. Mechanistically, the superior ZnO rate is consistent with our optical sector: a stronger band-edge absorption and exciton generation under sunlight, with radiative NBE channels largely quenched into charge separation pathways, features widely correlated with higher ROS yields (O2−•/OH•) and faster MB mineralization [27]. In contrast, Fe_3_O_4_ acts mainly as a dark adsorbent and a weak photoabsorber at these photon fluxes; intervalence/defect transitions and nonradiative relaxation limit ROS formation, explaining the lower k and RDR despite its high CE adsorption. Still, Fe_3_O_4_ composites can be valuable as magnetic supports or cocatalysts that facilitate charge shuttling when coupled to a wide-gap photoactive phase [149,150]. The ZnO/Fe_3_O_4_ nanocomposite achieves an intermediate k because the heterointerface both helps and hurts: interfacial fields and vacancy ladders (documented by PL) aid carrier separation, yet the effective optical cross-section of ZnO is diluted by the ferrite fraction, and some photocarriers drain nonproductively into Fe_3_O_4_’s defect manifold. This balance, improved separability and recyclability at a moderate kinetic penalty, is a common trade-off reported for Fe_3_O_4_–semiconductor systems engineered for sunlight MB degradation and easy magnetic recovery [146]. Two practical points reinforce data quality. First, adsorption correction (RDR = DR − AD) prevents overestimating performance, a best practice emphasized across recent MB photocatalysis reports [151]. Second, the linearity of the −ln(C/C_0_) plots over the operative window confirms the pseudo-first-order regime, in line with sunlight/visible studies that attribute rate control to surface reaction/ROS availability at low dye coverage [148]. Thus, ZnO remains the fastest photocatalyst in our conditions. However, the ZnO/Fe_3_O_4_ heterostructure provides a magnetically retrievable compromise with robust activity; Fe_3_O_4_ alone is dominated by sorption with limited photochemistry, findings that align with current literature and our structure–optics analysis. As a benchmark, Motelica et al. [25] reported nearly complete removal of MB (98% decolorization) using green-synthesized ZnO nanopowders under artificial visible-like irradiation, with comparable dye concentration and catalyst loading. Our sunlight-driven MB degradation efficiencies and rate constants for ZnO and magnetically recoverable ZnO/Fe_3_O_4_ therefore fall within the performance window of state-of-the-art, extract-assisted ZnO systems, while additionally offering magnetic recyclability and a detailed defect-chemistry analysis.

Further, when benchmarked against the literature (Table 4), our operating window, MB concentration = 10 mg L^−1^, photocatalyst weight PW = 10 mg in 50 mL (0.20 g L^−1^), natural sunlight and a total irradiation time of 180 min, places the adsorption-corrected outcomes in a realistic but relatively stringent regime. Many studies optimize apparent removal by using higher catalyst loadings, lower initial dye concentrations, or intense monochromatic UV sources, which can exaggerate performance metrics but are less representative of low-cost, field-operable conditions. In contrast, our configuration was deliberately chosen to mimic a simple batch treatment under broad-spectrum sunlight, with dark-adsorption baselines subtracted so that the reported RDR values reflect true photodegradation rather than a combination of adsorption and light-driven processes. Within this framework, ZnO stands out as highly competitive. Our RDR = 90% and k = 1.9 × 10^−2^ min^−1^ under natural sunlight match or slightly exceed other sunlight-driven reports for the same dye class at comparable doses (91.4–94%: [152,153]). At the same time, these values outperform several UV-driven cases conducted at higher dye concentration (DC) or shorter irradiation time (IT), such as 74% at 16 mg L^−1^ under UV [154] and 81% for methyl orange under UV [155]. This suggests that the defect structure and surface chemistry imparted by the biogenic route enable ZnO to maintain high activity even under less “idealized” illumination, while keeping the catalyst loading in a modest range. For Fe_3_O_4_, our adsorption-corrected RDR = 30% under sunlight may appear modest when compared with the 66.7% visible-light entry reported at higher PW and without explicit correction for adsorption [156]. However, the latter condition uses more catalyst and reports apparent decolorization, so part of the efficiency likely originates from strong dye uptake rather than complete mineralization. Our lower but carefully corrected value therefore provides a more conservative estimate of the intrinsic photoactivity of ferrite under sunlight, which is expected to be weaker than that of wide-band-gap ZnO. The ZnO/Fe_3_O_4_ composite naturally occupies an intermediate position. Its RDR = 65% lies below some UV/visible studies employing larger catalyst masses or more energetic excitation (79.7% under visible light: [157]; 98.2% under UV: [158]; 88.5% under visible light: [159]) and is comparable to mixed-dye situations such as rhodamine B removal (76.46% under UV: [160]). These differences are fully consistent with our ten-fold lower catalyst mass relative to several literature reports, the partial dilution of the ZnO optical cross-section by the ferrite fraction, and, again, the use of adsorption-corrected metrics. Overall, our kinetic constants for the composite and ferrite (0.7 and 0.4 × 10^−2^ min^−1^, respectively) fall squarely within the pseudo-first-order ranges previously reported for similar systems, reinforcing the robustness of the comparison and indicating that the present green-synthesized materials perform on par with, or better than, many conventional counterparts under more demanding operating conditions.

**Figure 10:**
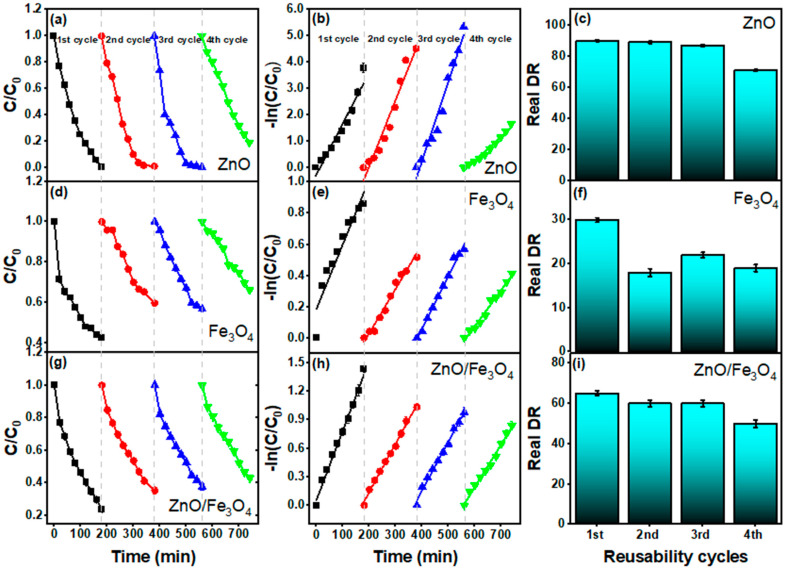
Reusability of ZnO, Fe_3_O_4_, and ZnO/Fe_3_O_4_ in sunlight-driven MB degradation over four consecutive cycles. (**a**,**d**,**g**) Normalized concentration profiles C/C_0_ versus time for cycles 1–4; (**b**,**e**,**h**) pseudo–first-order plots −ln(C/C_0_) showing linear fits within each cycle; (**c**,**f**,**i**) Real degradation rate (DR–AD) per cycle (adsorption-corrected using the dark control of Figure 6).

Figure 10 benchmarks reusability over four sunlight cycles after adsorption correction (DR–AD). For ZnO (Figure 10a–c), the C/C_0_(t) and −ln(C/C_0_) plots remain nearly parallel through cycles 1–3, with DR = 90%, 89%, 87% and k = 1.9, 2.5, and 3.0 × 10^−2^ min^−1^, respectively (Table 3). The modest decline in DR accompanied by stable pseudo–first-order linearity indicates preserved active sites and a reaction regime still governed by interfacial ROS generation rather than transport limitations, fully consistent with recent ZnO sunlight studies where kinetics follow −ln(C/C_0_) and activity is sustained across reuse [65,66,161]. A sharper drop appears in the 4th cycle (DR = 71%, k = 0.9 × 10^−2^ min^−1^), plausibly due to (i) partial dye/intermediate fouling, (ii) minor ZnO surface hydroxyl depletion/rearrangement, and (iii) incremental particle agglomeration, all commonly reported deactivation modes for oxide photocatalysts [26]. For Fe_3_O_4_ (Figure 10d–f), activity is lower and less stable (DR = 30%, 18%, 22%, 19%; k = 0.4–0.2 × 10^−2^ min^−1^). Magnetite primarily contributes dark adsorption (Figure 7) and acts as a weak photoabsorber; repeated operation can intensify surface spin disorder and promote surface coverage by aromatic residues, diminishing radical generation, trends widely noted for Fe_3_O_4_-centric photocatalysts unless coupled to a strong photoactive phase or co-catalyst [149,162]. The ZnO/Fe_3_O_4_ nanocomposite (Figure 10g–i) strikes a durable middle ground (DR = 65%, 60%, 60%, 50%; k = 0.7–0.4 × 10^−2^ min^−1^). The near-constant k through cycles 2–3 suggests that heterointerfacial fields and vacancy-rich junctions (established by PL) continue to assist charge separation, while magnetic recovery limits mass loss—behaviors repeatedly observed for ZnO–Fe_3_O_4_ and related magnetic heterostructures [149,162]. The gradual decline by cycle 4 likely stems from (i) partial masking of interfacial ZnO sites by persistent intermediates and (ii) slight reduction in specific interfacial area upon repeated drying/redispersion; both effects are reversible with mild solvent wash or low-temperature reactivation and are routinely documented in reuse studies of magnetic photocatalysts [162]. Our cyclic dataset confirms three practical points: (1) kinetics remain pseudo-first-order across cycles, validating a common rate law for MB photodegradation under sunlight [161], (2) ZnO delivers the highest per-cycle efficiency but lacks intrinsic magnetic separability, and (3) ZnO/Fe_3_O_4_ preserves a substantial fraction of its activity while enabling rapid magnetic recovery, aligning with contemporary designs that balance activity with recyclability for scalable water treatment.

**Figure 11:**
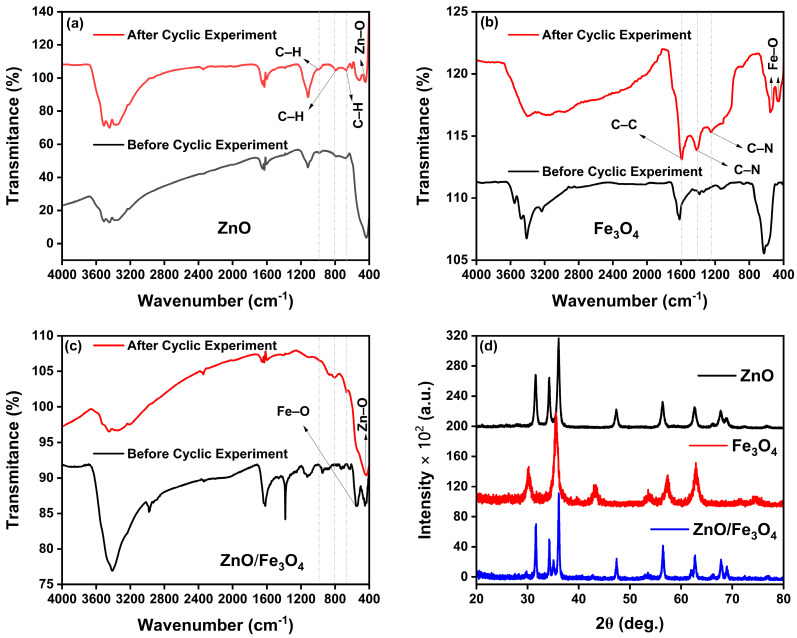
Post-cycle stability of the photocatalysts. FTIR spectra of (**a**) ZnO, (**b**) Fe_3_O_4_ and (**c**) ZnO/Fe_3_O_4_ before (black) and after (red) four photocatalytic cycles for MB degradation. (**d**) XRD patterns of ZnO, Fe_3_O_4_ and ZnO/Fe_3_O_4_ after cycling.

To clarify the origin of the performance drop observed in the cyclic tests, we carried out post-cycle FTIR and XRD analyses of all photocatalysts (Figure 11). The FTIR spectra recorded after four degradation cycles clearly show the presence of characteristic MB dye vibrations superimposed on the oxide bands. For ZnO and ZnO/Fe_3_O_4_ (Figure 11a,c), new bands appear at 671 cm^−1^ (out-of-plane C–H bending of the phenothiazine ring), 785 cm^−1^ (in-plane C–H bending), and 1038 cm^−1^ (in-plane C–H bending of the aromatic ring) [163], confirming that a fraction of MB or aromatic intermediates remains adsorbed on the surface. For Fe_3_O_4_ (Figure 11b), the MB fingerprint is even more evident: the band at 1612 cm^−1^ (C–C stretching of the aromatic ring) [164], together with the features at 1422 cm^−1^ (asymmetric C–N stretching and –N(CH_3_)_2_ vibrations) [163] and 1245 cm^−1^ (coupled ring/C–N modes associated with SERRS-active MB) [165], dominate the spectrum after cycling. The stronger MB signals in Fe_3_O_4_ are consistent with their higher adsorption capacity relative to bare ZnO and ZnO/Fe_3_O_4_, as observed in Figure 7, indicating that partial site blocking by strongly adsorbed dye/intermediates contributes to the moderate activity loss upon reuse rather than to structural degradation of the solids. Further, XRD patterns collected after the cyclic experiments (Figure 11d) show that all diffraction peaks for wurtzite ZnO and inverse-spinel Fe_3_O_4_ are preserved in position and shape, with no additional reflections attributable to secondary phases or crystalline by-products. The overall crystallinity remains essentially unchanged, aside from a slight attenuation of peak intensities that can be ascribed to surface coverage by residual organics. These results demonstrate that the crystal structures of ZnO, Fe_3_O_4_ and ZnO/Fe_3_O_4_ are stable under the photocatalytic conditions explored here, and that the gradual decrease in MB removal efficiency arises mainly from surface fouling by residual dye and intermediates rather than from phase transformation, dissolution or loss of magnetic properties.

**Figure 12:**
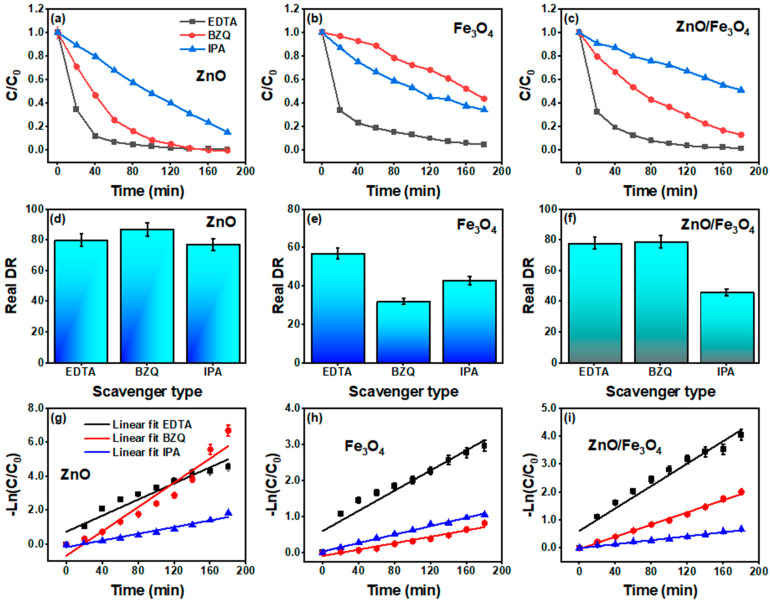
Elemental scavenger tests under natural sunlight for MB degradation using ZnO, Fe_3_O_4_, and ZnO/Fe_3_O_4_ nanomaterials. (**a**–**c**) normalized concentration profiles C/C_0_ versus time with EDTA (hole scavenger, $h^{+}$), BZQ (benzoquinone, O2−•), and IPA (isopropanol, OH•). (**d**–**f**) Real degradation rate (DR–AD) after adsorption correction. (**g**–**i**) pseudo–first-order kinetic plots −ln(C/C_0_) = kt with linear fits.

Figure 12 dissects the active species by adding EDTA, BZQ, and IPA while correcting for dark uptake (DR–AD). For ZnO (Figure 12a,d,g), the DR drops from 90% (ME) to 80% (EDTA), 87% (BZQ), and 77% (IPA) with the corresponding k values 2.3, 3.5, and 0.9 × 10^−2^ min^−1^. The largest inhibition by IPA signals a primary role of OH• radicals, with h^+^ and O2−•, acting secondarily, consistent with sunlight ZnO systems where surface –OH/adsorbed H_2_O rapidly convert photogenerated h^+^/e^−^ into OH•/O2−• species that drive MB mineralization. Recent ZnO studies using the same quenchers report the same hierarchy (OH• > h^+^ ~ O2−•) [161,166,167]. For Fe_3_O_4_ (Figure 12b,e,h), the picture shifts: DR changes from 30% (ME) to 32% (BZQ), 43% (IPA), and 57% (EDTA) with small k values (Table 3). The strongest suppression by BZQ indicates that O2−• is the key oxidant in magnetite-only runs, while ^•^OH/h^+^ contributions are weaker, aligning with reports that iron oxides favor one-electron pathways and superoxide-mediated dye attack under solar excitation. The atypically higher DR with EDTA is not unprecedented for Fe-oxide surfaces: EDTA can complex Fe^3+^ and promote ligand-to-metal charge transfer or alter surface charge, occasionally modulating, not purely quenching, hole chemistry, as observed in iron-oxide photocatalysis and related ROS studies [168,169]. The ZnO/Fe_3_O_4_ nanocomposite (Figure 12c,f,i) retains 65% → 78% (EDTA), 79% (BZQ), and 46% (IPA) with k = 2.0, 1.1, and 0.4 × 10^−2^ min^−1^. The pronounced inhibition by IPA again identifies OH• as the dominant oxidant at the heterointerface, whereas EDTA/BZQ produce modest changes, consistent with a Z-/S-scheme–like separation where h^+^ on ZnO efficiently generates OH• while Fe_3_O_4_ serves mainly as an electron sink/transport hub that moderates O2−• formation. Comparable Fe_3_O_4_–ZnO heterostructures show similar ROS fingerprints and kinetics under sunlight [12]. In this regard, our radical-trapping results show that for OH• and O2−• to emerge as the dominant reactive species, while hole scavenging has a comparatively weaker effect, electrons must remain in a CB sufficiently negative to reduce O_2_ and holes must reside in a VB sufficiently positive to oxidize H_2_O/OH^−^. This redox pattern is a key diagnostic often used to distinguish S-/Z-scheme pathways from conventional type-II junctions in oxide-based photocatalysts [35,45,133]. Combined with the fact that ZnO/Fe_3_O_4_ delivers adsorption-corrected degradation and rate constants comparable to or better than ZnO under sunlight, despite containing only half the ZnO mass, this behavior is inconsistent with a conventional type-II junction that sacrifices redox strength for separation. Instead, it closely mirrors the synergistic response observed in Fe_3_O_4_/ZnO and other magnetically recoverable ZnO-based heterostructures, which have been rationalized using Z-scheme or S-scheme charge-transfer pathways [9,48,50]. Collectively, the scavenger assays corroborate our optical diagnosis (interfacial vacancy ladders and band bending) by showing that hydroxyl radicals govern MB removal in ZnO and ZnO/Fe_3_O_4_, while superoxide dominates in Fe_3_O_4_; the pseudo–first-order fits in Figure 12g–i confirm that mechanistic assignments carry through to the rate law over the operative conversion window.

**Figure 13:**
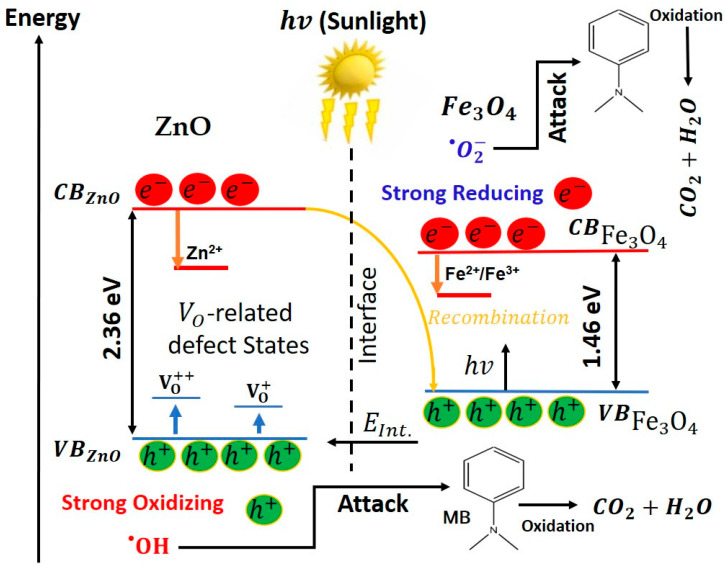
Schematic illustration of the proposed S/Z-scheme-like charge-transfer mechanism in the ZnO/Fe_3_O_4_ heterojunction under sunlight.

Figure 13 schematically summarizes how structure and defect chemistry determine the photocatalytic response of the ZnO, Fe_3_O_4_ and ZnO/Fe_3_O_4_ nanomaterials. Linking activity to physics, our data indicate that defect chemistry, interfacial band bending, particle size/morphology and trace surface impurities act synergistically to set the radical budget that ultimately degrades MB. PL signatures in ZnO and ZnO/Fe_3_O_4_ (broad green–yellow band at 490–560 nm) point to abundant $V_{O}/V_{O}^{++}$ states; contemporary analyses show that such oxygen vacancies can pin the surface Fermi level, promote H_2_O/O_2_ adsorption and activation, extend carrier lifetimes and accelerate ROS formation under solar excitation, precisely the behaviour inferred from the strong tert-butanol/IPA inhibition observed in our scavenger assays [170,171]. At the ZnO/Fe_3_O_4_ junction, coherent or semi-coherent contacts generate internal electric fields that drive an S/Z-scheme-like separation: low-energy electrons in CB_ZnO_ recombine with low-energy holes in ${VB}_{{Fe}_{3}O_{4}}$, whereas the most oxidizing holes are retained on VB_ZnO_ (where they convert H_2_O/OH^−^ into OH•), and the most reducing electrons percolate into ${CB}_{{Fe}_{3}O_{4}}$ to activate O_2_ into O2−•. Recent Fe_3_O_4_/ZnO heterostructures report a very similar band alignment, defect-assisted visible absorption and recombination suppression as the key ingredients behind enhanced sunlight activity [12]. Particle size and morphology further bias the outcome: shrinking ZnO domains shortens diffusion paths and increases the density of reactive surface –OH groups, while anisotropic facets concentrate local fields and preferentially localize holes at adsorption-favoured sites, morphology–activity couplings repeatedly documented in modern ZnO photocatalysis [172,173]. Residual carbon from the green route may act as a benign photosensitizer or electron mediator that broadens visible-light uptake and facilitates interfacial charge transport, whereas thick carbon shells behave as recombination reservoirs; controlled carbon–ZnO coupling is therefore beneficial only within a narrow coverage window [174,175]. On the ferrite side, Fe_3_O_4_ alone shows limited photo-response (low k, low RDR) because intervalence transitions and surface spin disorder favor nonradiative relaxation; enhancing its role typically requires plasmonic/2D co-catalysts or intimate junctions that channel electrons efficiently, designs validated in recent iron-oxide composites for MB removal [150]. Finally, the adsorption microphysics set the stage for true catalysis: excessive dark uptake can mask kinetics and promote site blocking, whereas moderate, well-distributed coverage maximizes photon-to-ROS conversion; current assessments emphasize rigorous adsorption correction (DR–AD) and caution that “selective” scavengers can perturb multiple pathways [176,177], exactly the protocols we adopted in Figure 7, Figure 9, Figure 10 and Figure 12. Therefore, high oxygen vacancy density on ZnO, field-sustaining ZnO/Fe_3_O_4_ interfaces, nanoscale domain control, and tuned surface carbon/charge collectively tip the balance toward OH•-dominated routes in ZnO and the composite, while Fe_3_O_4_ alone leans on O2−• chemistry with subdued solar harvesting, an integrated structure–property–function picture consistent with the latest literature [15].

The photocatalytic activity of these nanomaterials arises from the following sequence of processes. Under natural sunlight, the sequence begins with photoexcitation, where photons promote electrons from the valence band (VB) to the conduction band (CB), Equations (2) and (3).

(2) $$ZnO+hv\to e_{CB}^{-}\left(ZnO\right)+h_{VB}^{+}\left(ZnO\right)$$

(3) $${Fe}_{3}O_{4}+hv\to e_{CB}^{-}\left({Fe}_{3}O_{4}\right)+h_{VB}^{+}\left({Fe}_{3}O_{4}\right)$$

In the heterostructure, band offsets and the built-in interfacial field drive an S/Z-scheme–like recombination of the low-energy carriers (Equation (4)), retaining the high-energy pair $h_{VB}^{+}\left(ZnO\right)$ and $e_{CB}^{-}\left({Fe}_{3}O_{4}\right)$ for redox chemistry.

(4) $$\left({Fe}_{3}O_{4}\right)\to heat$$

The oxidation branch starts as photoholes on ZnO are trapped at surface hydroxyls/water to generate hydroxyl radicals, as shown by Equations (5) and (6) [36,178].

(5) eCB−ZnO+hVB+hVB+ZnO+H2O→OH•+H+

(6) hVB+ZnO+OH−→OH•

Concurrently, the reduction branch proceeds when electrons reduce dissolved oxygen (Equation (7)). Superoxide then undergoes a proton–electron cascade that amplifies the radical pool, as shown by Equations (8)–(10) [178]:

(7) eCB−ZnO/Fe3O4+O2→O2−•

(8) O2−•+H+→HO2•

(9) HO2•+e−→HO2−

(10) $${HO}_{2}^{-}+H^{+}\to H_{2}O_{2}$$

and

(11) H2O2+hvor e−→2OH•

On Fe_3_O_4_ surfaces this peroxide can also be activated through photo-Fenton-like steps that complement the electron pathway, Equations (12) and (13), thereby regenerating Fe^2+^ and sustaining ROS production at the interface.

(12) Fe2++H2O2→Fe3++OH•+OH−

(13) Fe3++H2O2→Fe2++HO2•+H+

In parallel, oxygen-vacancy states on ZnO accelerate hole trapping and water activation; vacancy-assisted pathways cooperate with superoxide to enrich OH• and $H_{2}O_{2}$ formation, as seen in Equation (14).

(14) hVO+/VO+++(ZnO)+O2−•+2H2O→2OH•+H2O2

The resulting radical budget, dominated by OH• on ZnO (and at ZnO/Fe_3_O_4_ junctions) and by O2−• on Fe_3_O_4_, drives stepwise N-demethylation, aromatic-ring opening, and ultimate mineralization of MB hazardous molecules, as illustrated in Equation (15).

(15) MB+OH•/O2−•→intermediates→CO2+H2O+inorganics ions

Therefore, oxygen vacancies boost hole utilization and water oxidation, while the heterojunction’s internal field suppresses bulk recombination and channels electrons into Fe_3_O_4_; the full photoexcitation → radical generation → pollutant oxidation chain thus proceeds efficiently, and the composite remains readily recyclable owing to its magnetic recoverability.

## 4. Conclusions

In summary, we demonstrate a bioengineered, hydrothermally coupled ZnO/Fe_3_O_4_ heterostructure that unites phase-pure wurtzite and inverse-spinel domains into a mechanically intact, defect-tunable interface optimized for sunlight photocatalysis. Rietveld/TEM show biphasic integrity with asymmetric size–strain partitioning (ZnO coarsening and strain relief; Fe_3_O_4_ retained nanoscale with slight strain increase), while PL reveals NBE quenching in ZnO and reweighted green–yellow bands from vacancy ladders, optical signatures of interfacial fields and vacancy-rich surfaces. Under natural sunlight at a realistic operating window (MB = 10 mg L^−1^, catalyst loading = 10 mg in 50 mL), ZnO reaches an adsorption-corrected removal degree RDR of 90% with k = 1.9 × 10^−2^ min^−1^, the ZnO/Fe_3_O_4_ composite attains RDR = 65% with k = 0.7 × 10^−2^ min^−1^, and Fe_3_O_4_ alone yields RDR = 30% with k = 0.4 × 10^−2^ min^−1^, placing the green-synthesized materials on par with or above many state-of-the-art systems that use higher catalyst doses or artificial irradiation. In recyclability tests, ZnO and ZnO/Fe_3_O_4_ preserve their pseudo–first-order behavior over four cycles, with MB removal decreasing modestly from 90 to 71% and from 65 to 50%, respectively, while the composite remains magnetically recoverable within seconds, evidencing a robust compromise between catalytic activity and ease of separation. Scavenger assays identify OH•-dominated pathways for ZnO and ZnO/Fe_3_O_4_ and O2−•-centric routes for Fe_3_O_4_, consistent with an S/Z-scheme-like mechanism that preserves high-energy $h_{VB}^{+}\left(ZnO\right)$/$e_{CB}^{-}\left({Fe}_{3}O_{4}\right)$ pairs while suppressing bulk recombination. This provides a clear heuristic: high oxygen-vacancy density on ZnO, interfacial band bending at the ZnO/Fe_3_O_4_ junction, and controlled nanoscale domain sizes jointly set the radical budget and define a design rulebook for defect-engineered, magnetically retrievable photocatalysts operating under real sunlight and modest loadings. Beyond MB, the structure–property rules uncovered here, defect engineering, interfacial band alignment, and size/morphology control, offer a transferable toolkit for other oxide–ferrite systems. Looking forward, catalytic efficiency can be further improved by (i) systematically tuning the ZnO/Fe_3_O_4_ mass ratio and defect density, (ii) integrating additional green co-catalysts (e.g., biochar or carbon dots) to extend visible-light harvesting, and (iii) applying the present platform to more complex pollutants and real effluents. These directions position the present bioengineered ZnO/Fe_3_O_4_ heterostructure not only as a competitive photocatalyst for MB removal, but also as a scalable starting point for next-generation, sustainable water-remediation technologies.

## Figures and Tables

**Table 1:** Rietveld-refined structural parameters from XRD for ZnO, Fe_3_O_4_, and the ZnO/Fe_3_O_4_ nanocomposite. Listed are the goodness-of-fit indices (Rwp, χ^2^), crystal structure and space group (ZnO: P6_3_mc; Fe_3_O_4_: Fd$\bar{3}$m), lattice parameters (a = b, c for hexagonal; a = b = c for cubic), unit-cell volume (V), theoretical density (σ), XRD crystallite size (D_XRD_) (Scherrer), and microstrain (ε). For the composite, values are given as ZnO/Fe_3_O_4_; “***” indicates not applicable.

Parameter	Samples		
	ZnO	Fe_3_O_4_	ZnO/Fe_3_O_4_
Rwp	7.96	11	8.24
χ^2^	1.24	1.17	1.1
Crystal structure	Hexagonal	Cubic	Hexagonal/Cubic
Space Group	P6_3_mc	Fd$\bar{3}$m	P6_3_mc/Fd$\bar{3}$m
a = b (Å)	3.2538	***	3.2513
a = b = c (Å)	***	8.3479	8.3840
c (Å)	5.2130	***	5.2090
V (Å^3^)	47.80	581.75	47.69/589.32
σ (g/cm^3^)	5.72	5.65	5.67/6.11
D_DRX_ (nm)	15.2	7.9	20.8/8.7
ε (%)	41	23	24/29

**Table 2:** Bandgap luminescence and deconvoluted PL peak positions for ZnO, Fe_3_O_4_, and ZnO/Fe_3_O_4_ heterostructures with defect-related assignments and literature sources; acronyms and symbols: NBE = near band-edge emission (free/bound excitons or band-to-band), CB/VB = conduction/valence band, $h^{+}$ = hole, $V_{O}/V_{O}^{+}/V_{O}^{++}\;$ = oxygen vacancy in neutral/singly/doubly ionized states, $O_{i}$ = oxygen interstitial, ${Zn}_{i}$ = zinc interstitial, $V_{Zn}$ = zinc vacancy, $V_{Fe}$ = iron vacancy (charge state context-dependent), ${NBE}_{ZnO}/{NBE}_{{Fe}_{3}O_{4}}$ = band-edge emission attributed to the indicated phase within the heterostructure, MBE = mid-gap (deep-level) emission not tied to a single discrete center, and $V_{O}^{+/++}@S$ = surface-trap level(s) associated with singly/doubly ionized oxygen-vacancy centers.

Sample	Parameters		
	Peak Position (nm)	Defect	References
**ZnO**	399	NBE	[91,92,93]
	423	CB → $V_{Zn}$, ${Zn}_{i}\to VB$	[94,95,96]
	440	${Zn}_{i}$, $O_{i}$, $V_{Zn}^{+}$	[97,98,99,100]
	462	${Zn}_{i}$, $V_{O}$, $V_{O}^{+}\to VB$	[101,102]
	487	$V_{O}$, $V_{O}^{+},O_{i}$	[97,103,104]
	517	$V_{O}^{+}$	[100,105,106]
	562	$V_{O}^{+}$	[107,108,109]
	567	$V_{O}^{++}$	[110]
**Fe_3_O_4_**	307	MBE	[111]
	354	MBE, $V_{O}$	[112]
	398	NBE, $h^{+}$	[55,113]
	465	$V_{O}$	[102,114]
	520	$V_{O}^{+/++}@S$	[115]
	585	$V_{O}$	[42,114,116]
**ZnO/Fe_3_O_4_**	380	${NBE}_{ZnO}$	[91,92,93,97]
	394	${NBE}_{{Fe}_{3}O_{4}}$, $h^{+}$	[55,117]
	418	CB → $V_{Zn}$, ${Zn}_{i}\to VB$	[94,95,96]
	452	${Zn}_{i}\to V_{Zn}$	[118,119]
	490	$V_{O}$, $V_{O}^{+},O_{i},\;V_{O}^{++}$	[97,120,121]
	525	$V_{O}^{+},\;{\;V}_{O}^{+/++}@S$	[100,106,115]
	560	$V_{O}^{+}$, $V_{O}^{++}$	[116,122,123]
	620	$O_{i}$, ${Zn}_{i}\to O_{i}$	[124,125,126]
	650	$V_{O},\;V_{Fe}$	[127,128]

**Table 3:** Summary of photodegradation metrics for MB using ZnO, Fe_3_O_4_, and ZnO/Fe_3_O_4_ under natural sunlight and dark control. Results are organized by control experiment (CE), main experiment (ME), cyclic experiment (CyE; 1st–4th reuse), and scavenger experiment (SE; EDTA, BZQ, IPA). AD = adsorption rate in the dark; RDR = real degradation rate = (degradation under sunlight, DR)—(AD from CE); k = pseudo-first-order kinetic constant (min^−1^). Error bars, where reported, denote the standard deviation of triplicates.

Experiments/Parameters			Samples		
			ZnO	Fe_3_O_4_	ZnO/Fe_3_O_4_
CE		AD (%)	10	49	14
ME		RDR (%)	90	30	65
		k (min^−1^)	${1.9\times 10}^{-2}$	${0.4\times 10}^{-2}$	${0.7\times 10}^{-2}$
CyE	1st	DR (%)	90	30	65
		k (min^−1^)	${1.9\times 10}^{-2}$	${0.4\times 10}^{-2}$	${0.7\times 10}^{-2}$
	2nd	DR (%)	89	18	60
		k (min^−1^)	${2.5\times 10}^{-2}$	${0.3\times 10}^{-2}$	${0.5\times 10}^{-2}$
	3rd	DR (%)	87	22	60
		k (min^−1^)	${3.0\times 10}^{-2}$	${0.3\times 10}^{-2}$	${0.5\times 10}^{-2}$
	4th	DR (%)	71	19	50
		k (min^−1^)	${0.9\times 10}^{-2}$	${0.2\times 10}^{-2}$	${0.4\times 10}^{-2}$
SE	EDTA	DR (%)	80	57	78
		k (min^−1^)	${2.3\times 10}^{-2}$	${1.3\times 10}^{-2}$	${2.0\times 10}^{-2}$
	BZQ	DR (%)	87	32	79
		k (min^−1^)	${3.5\times 10}^{-2}$	${0.4\times 10}^{-2}$	${1.1\times 10}^{-2}$
	IPA	DR (%)	77	43	46
		k (min^−1^)	${0.9\times 10}^{-2}$	${0.6\times 10}^{-2}$	${0.4\times 10}^{-2}$

**Table 4:** Benchmarking the photocatalytic performance of ZnO, ZnO/Fe_3_O_4_ and Fe_3_O_4_ systems reported in the literature. Comparative overview of dye type (DT), photocatalyst weight (PW, mg), dye concentration (DC, mg L^−1^), irradiation time (IT, min), light source (LS), and maximum photocatalytic efficiency (MPE, %) used to contextualize the present ZnO-based photocatalyst within the broader state of the art.

Photocatalyst	DT	PW	DC	IT	LS	MPE	Reference
	-	mg	Mg·L^−1^	min	-	%	-
ZnO	methylene blue	100	40	180	sunlight	91.4	[152]
ZnO	methylene blue	20	10	150	sunlight	94	[153]
ZnO	methylene blue	20	16	180	UV light	74	[154]
ZnO	methyl orange	150	20	100	UV light	81	[155]
Fe_3_O_4_	methylene blue	40	10	180	visible light	66.7	[156]
ZnO/Fe_3_O_4_	methylene blue	45	100	150	visible light	79.7	[157]
ZnO/Fe_3_O_4_	methyl orange	100	10	40	UV light	98.2%	[158]
ZnO/Fe_3_O_4_	methylene blue	200	100	120	visible light	88.5	[159]
ZnO/Fe_3_O_4_	rhodamine B	60	24	180	UV light	76.46	[160]

## Data Availability

The data presented in this study are available from the corresponding author upon reasonable request.
